# Complete genome sequence of *Acidaminococcus fermentans* type strain (VR4^T^)

**DOI:** 10.4056/sigs.1002553

**Published:** 2010-07-29

**Authors:** Yun-Juan Chang, Rüdiger Pukall, Elizabeth Saunders, Alla Lapidus, Alex Copeland, Matt Nolan, Tijana Glavina Del Rio, Susan Lucas, Feng Chen, Hope Tice, Jan-Fang Cheng, Cliff Han, John C. Detter, David Bruce, Lynne Goodwin, Sam Pitluck, Natalia Mikhailova, Konstantinos Liolios, Amrita Pati, Natalia Ivanova, Konstantinos Mavromatis, Amy Chen, Krishna Palaniappan, Miriam Land, Loren Hauser, Cynthia D. Jeffries, Thomas Brettin, Manfred Rohde, Markus Göker, James Bristow, Jonathan A. Eisen, Victor Markowitz, Philip Hugenholtz, Nikos C. Kyrpides, Hans-Peter Klenk

**Affiliations:** 1 DOE Joint Genome Institute, Walnut Creek, California, USA; 2 Oak Ridge National Laboratory, Oak Ridge, Tennessee, USA; 3 DSMZ – German Collection of Microorganisms and Cell Cultures GmbH, Braunschweig, Germany; 4 Los Alamos National Laboratory, Bioscience Division, Los Alamos, New Mexico, USA; 5 Biological Data Management and Technology Center, Lawrence Berkeley National Laboratory, Berkeley, California, USA; 6 HZI – Helmholtz Centre for Infection Research, Braunschweig, Germany; 7 University of California Davis Genome Center, Davis, California, USA; * Corresponding author: Hans-Peter Klenk; **Keywords**: anaerobic, mesophile, diplococcus, gastrointestinal tract, trans-aconitate degradation, glutamate fermentation, *Acidaminococcaceae*, *Selenomonadales*,* Negativicutes*, GEBA

## Abstract

*Acidaminococcus fermentans* (Rogosa 1969) is the type species of the genus *Acidaminococcus*, and is of phylogenetic interest because of its isolated placement in a genomically little characterized region of the *Firmicutes*. *A. fermentans* is known for its habitation of the gastrointestinal tract and its ability to oxidize *trans*-aconitate. Its anaerobic fermentation of glutamate has been intensively studied and will now be complemented by the genomic basis. The strain described in this report is a nonsporulating, nonmotile, Gram-negative coccus, originally isolated from a pig alimentary tract. Here we describe the features of this organism, together with the complete genome sequence, and annotation. This is the first complete genome sequence of a member of the family *Acidaminococcaceae*, and the 2,329,769 bp long genome with its 2,101 protein-coding and 81 RNA genes is part of the *** G****enomic* *** E****ncyclopedia of* *** B****acteria and* *** A****rchaea * project.

## Introduction

Strain VR4^T^ (= DSM 20731 = ATCC 25085 = CCUG 9996) is the type strain of the species *Acidaminococcus fermentans*, and the type species of the genus *Acidaminococcus* [1,2]. *A. fermentans* was originally isolated by Fuller from a pig alimentary tract [3] and included in the *nomina incertae sedis.* It was subsequently characterized and classified in 1969 as type strain of the then-novel genus *Acidaminococcus* [1]. An emendation of the description of *A. fermentans* was provided by Cook *et al.* in 1994 [4]. Originally, the principal physiological and taxonomically distinctive feature of the strain was its ability to use amino acids as sole source of energy for growth anaerobically [1]. Three accompanying strains (VR7, VR11, VR14) from the alimentary tract of the same pig were reported and deposited in the ATCC (American Type Culture Collection) [3]. Closely related strains belonging to the species have been isolated from humans (EF060089-91, >99.9% sequence identity) [5] and from cow rumen [4]. Several uncultured clones were isolated from human fecal samples (DQ904734, DQ904735 and DQ904837, 99.8%) [6], and from rabbit cecum (EF445291, 99.8%) [7]. The type strain of the only other species in the genus, *A. intestini* [5] shows 95.8% 16S rRNA sequence identity with strain VR4^T^, whereas the type species of the other genera of the *Acidaminococcaceae* are less than 91.7% identical [8]. Of the many publicly available human gut metagenomes only one (BAAV01001815, 96.1%) [9] contained a highly similar 16S rRNA gene sequence, whereas none of the environmental genomic surveys indicated any moderately related phylotypes, shedding doubt on a wide-spread occurrence of members of the species *A. fermentans* (as of February 2010). Here we present a summary classification and a set of features for *A. fermentans* VR4^T^, together with the description of the complete genomic sequencing and annotation.

## Classification and features

**Figure 1:**
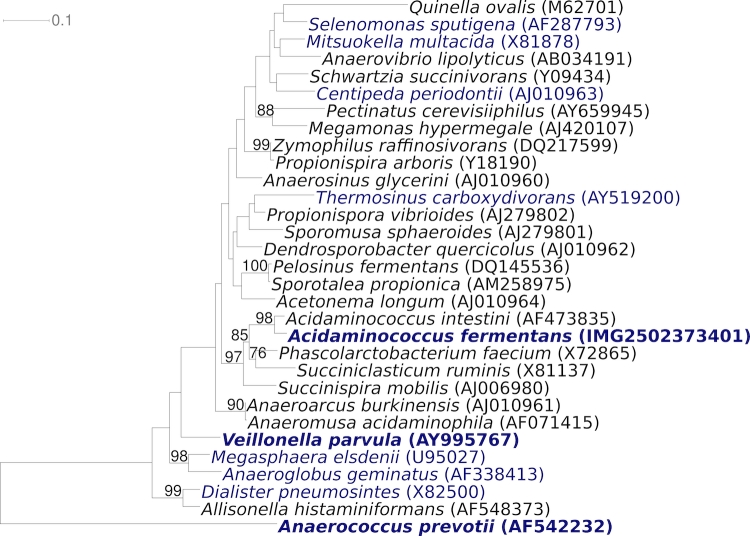
Phylogenetic tree highlighting the position of *A. fermentans* VR4^T^ relative to the other type strain within the genus *Acidaminococcus* and the type strains of the other genera within the family *Acidaminococcaceae*. The tree was inferred from 1,348 aligned characters [10,11] of the 16S rRNA gene sequence under the maximum likelihood criterion [12] and rooted with the type strain of *Anaerococcus prevotii*, a member of the neighboring family *Peptococcaceae*. The branches are scaled in terms of the expected number of substitutions per site. Numbers above branches are support values from 1,000 bootstrap replicates [13] if larger than 60%. Lineages with type strain genome sequencing projects registered in GOLD [14] are shown in blue, published genomes in bold and include. the recently published GEBA genome from *A. prevotii* [15] and *Veillonella parvula* [16].

Figure 1 shows the phylogenetic neighborhood of *A. fermentans* VR4^T^ in a 16S rRNA based tree. The sequences of the six copies of the16S rRNA gene in the genome of strain VR4^T^ differ from each other by up to five nucleotides, and differ by up to seven nucleotides from the previously published 16S rRNA sequences from DSM 20731 (X78017, X77951), which contain two ambiguous base calls.

**Figure 2:**
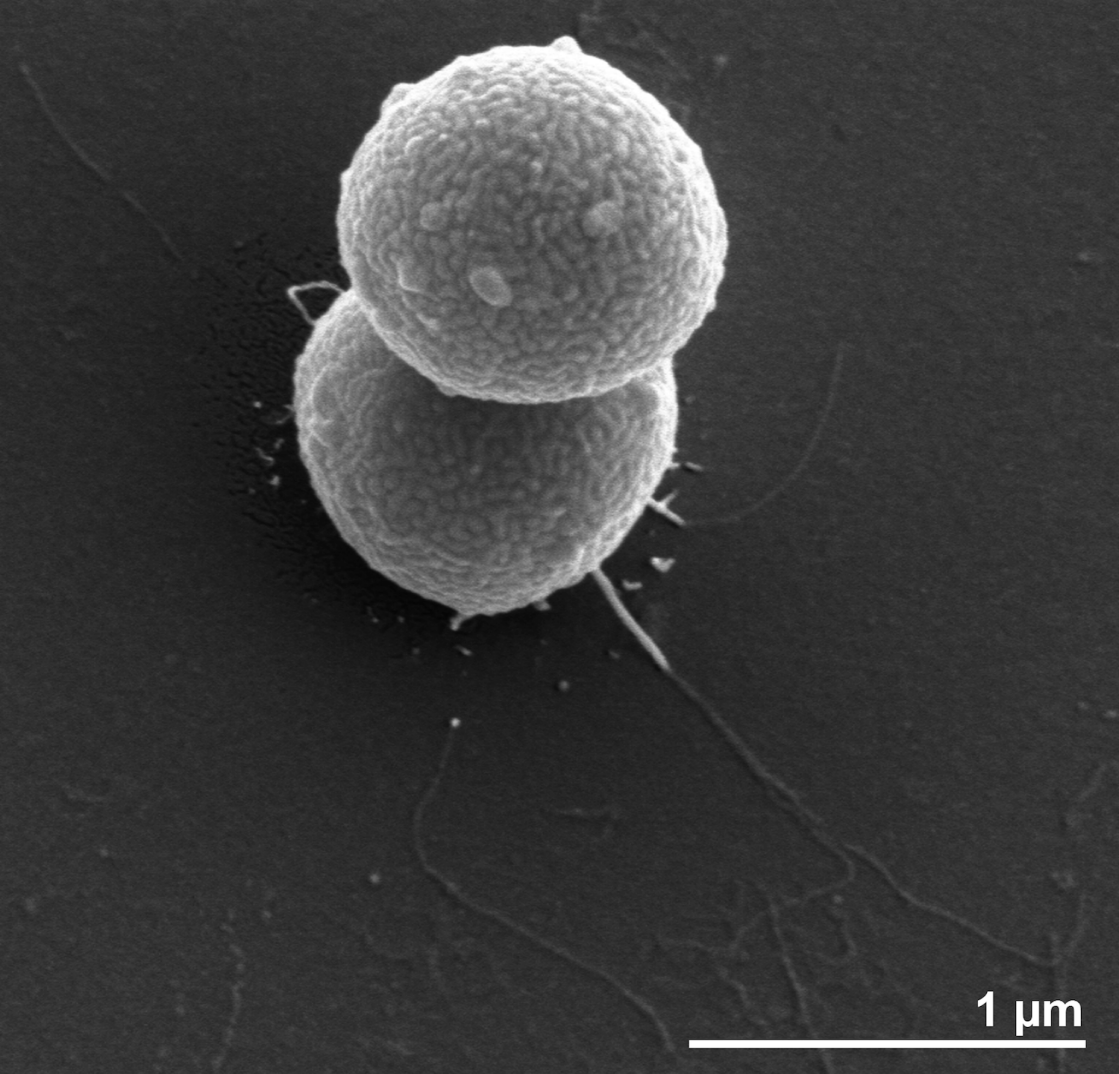
Scanning electron micrograph of *A. fermentans* strain VR4^T^

Cells of *A. fermentans* strain VR4^T^ often occur as oval or kidney-shaped diplococci that are 0.6 to 1.0 μm in diameter [1] (Table 1 and Figure 2). The organism is anaerobic, nonsporulating, nonmotile, chemoorganotrophic, and Gram-negative [1]. Its optimum growth temperature is 30 to 37°C, over a pH range from 6.2 to 7.5, with an optimum at 7.0 [1]. Weak or no growth occurs at 25 and 45°C.

**Table 1:** Classification and general features of *A. fermentans* VR4^T^ according to the MIGS recommendations [17]

**MIGS ID**	**Property**	**Term**	**Evidence code**
	Current classification	Domain *Bacteria* Phylum *Firmicutes* Class *Negativicutes* Order *Selenomonadales* Family *Acidaminococcaceae* Genus *Acidaminococcus* Species *Acidaminococcus fermentans* Type strain VR4	TAS [18] TAS [19,20] TAS [21] TAS [21] TAS [21] TAS [1,2,4,5] TAS [1] TAS [1,2,4]
	Gram stain	negative	TAS [1]
	Cell shape	oval; kidney shaped diplococci	TAS [1]
	Motility	nonmotile	TAS [1]
	Sporulation	non-sporulating	TAS [1]
	Temperature range	mesophilic	TAS [1]
	Optimum temperature	30-37°C	TAS [1]
	Salinity	moderate	TAS [1]
MIGS-22	Oxygen requirement	anaerobic	TAS [1]
	Carbon source	glutamate	TAS [1]
	Energy source	glutamate, citrate, trans-aconitate	TAS [1,4]
MIGS-6	Habitat	gastrointestinal tract of homothermic animals	TAS [22]
	pH	6.2–7.5, optimum 7.0	TAS [23]
MIGS-15	Biotic relationship	free-living	TAS [1]
MIGS-14	Pathogenicity	pathogenic for humans	TAS [24]
	Biosafety level	2	TAS [24]
	Isolation	*Sus scrofa*, alimentary tract	TAS [1,3]
MIGS-4	Geographic location	not reported	NAS
MIGS-5	Sample collection time	about 1966	TAS [3]
MIGS4.1 MIGS-4.2	Latitude Longitude	not reported	NAS
MIGS-4.3	Depth	not reported	NAS
MIGS-4.4	Altitude	not reported	NAS

Evidence codes - IDA: Inferred from Direct Assay (first time in publication); TAS: Traceable Author Statement (i.e., a direct report exists in the literature); NAS: Non-traceable Author Statement (i.e., not directly observed for the living, isolated sample, but based on a generally accepted property for the species, or anecdotal evidence). These evidence codes are from the Gene Ontology project [25]. If the evidence code is IDA, then the property was directly observed for a live isolate by one of the authors or an expert mentioned in the acknowledgements.

*A. fermentans* thrives mainly by glutamate fermentation *via* the 2-hydroxyglutarate pathway in the intestinal tract of homeothermic animals [1,25], utilizing glutamate, citrate, and *trans*-aconitate as sole energy sources, in the presence of sodium [4,26,27]. Ammonia, acetate, butyrate, and hydrogen are the main end products of growth [1,4]. Transport and catabolism of these substrates are dependent on a sodium motive force as a method of membrane energetics [27,28]. The fermentation of *trans*-aconitate in strain VR4^T^ takes a different pathway, which is *via* citrate, oxaloacetate, and pyruvate, producing CO_2_, acetate, butyrate and hydrogen [26]. Unlike the citrate uptake, the aconitate uptake may adopt two mechanisms: a citrate/aconitate carrier with low affinity for sodium and an aconitate carrier with high affinity for sodium [29].

### Chemotaxonomy

*A. fermentans* VR4^T^ contains meso-diaminopimelic acid in the cell wall; extracts from whole cells contain galactose, glucose and ribose. Menaquinones and ubiquinones are absent; phospholipids present are disphosphatidyl-glycerol, phosphatidylethanolamine and an additional phospholipid, possibly phosphatidylcholine [30]. Major fatty acids (>5% of total) are C_16:1 cis-7_ (30%), C_14:0-3OH_ (15.8%), C_18:1 cis-11_ (10.2%), C_12:0_ (9.8%), C_16:1 cis-7_ (9.5%), and C_18:1 cis-9_ (5.8%) [30]. Monoenoic unsaturated phospholipid fatty acids (PLFAs) are dominant, indicating a Gram-negative bacterium, branched monoenoic or mid-branched saturated PLFAs as biomarker for anaerobic respiration is also prominent [31].

## Genome sequencing and annotation

### Genome project history

This organism was selected for sequencing on the basis of its phylogenetic position [32], and is part of the *** G****enomic* *** E****ncyclopedia of* *** B****acteria and* *** A****rchaea * project [33]. The genome project is deposited in the Genomes OnLine Database [14] and the complete genome sequence in GenBank. Sequencing, finishing and annotation were performed by the DOE Joint Genome Institute (JGI). A summary of the project information is shown in Table 2.

**Table 2:** Genome sequencing project information

**MIGS ID**	**Property**	**Term**
MIGS-31	Finishing quality	Finished
MIGS-28	Libraries used	One genomic 8kb pMCL200 library, one 454 pyrosequence library and one Illumina library
MIGS-29	Sequencing platforms	ABI3730, 454 Titanium, Illumina GA
MIGS-31.2	Sequencing coverage	5× Sanger; 58.3× pyrosequence
MIGS-30	Assemblers	Newbler version 2.0.0-PostRelease-11/04/2008, phrap
MIGS-32	Gene calling method	Prodigal, GenePRIMP
	INSDC ID	CP001859
	Genbank Date of Release	January 19, 2010
	GOLD ID	Gc01187
	NCBI project ID	33685
	Database: IMG-GEBA	2502171195
MIGS-13	Source Material Identifier	DSM 20731
	Project relevance	Tree of Life, GEBA

### Growth conditions and DNA isolation

*A. fermentans* VR4^T^, DSM 20731, was grown under strictly anaerobic conditions in DSMZ medium 414 [34] at 37°C. DNA was isolated from 1-1.5 g of cell paste using Qiagen Genomic 500 DNA Kit (Qiagen, Hilden, Germany) following the manufacturer's instructions with modification st/LALMP for cell lysis according to Wu *et al*. [33].

### Genome sequencing and assembly

The genome was sequenced using a combination of Sanger and 454 sequencing platforms. All general aspects of library construction and sequencing can be found at http://www.jgi.doe.gov/. 454 Pyrosequencing reads were assembled using the Newbler assembler version 2.0.0-PostRelease-11/04/2008 (Roche). Large Newbler contigs were broken into 2,561 overlapping fragments of 1,000 bp and entered into the final assembly as pseudo-reads. The sequences were assigned quality scores based on Newbler consensus q-scores with modifications to account for overlap redundancy and to adjust inflated q-scores. A hybrid 454/Sanger assembly was made using the parallel phrap assembler (High Performance Software, LLC). Possible mis-assemblies were corrected with Dupfinisher or transposon bombing of bridging clones [35]. Gaps between contigs were closed by editing in Consed, custom primer walk or PCR amplification. A total of 256 Sanger finishing reads were produced to close gaps, to resolve repetitive regions, and to raise the quality of the finished sequence. Illumina reads were used to improve the final consensus quality using an in-house developed tool (the Polisher). The error rate of the completed genome sequence is less than 1 in 100,000. Together all sequence types provided 63.3× coverage of the genome. The final assembly contains 22,991 Sanger and 557,705 pyrosequencing reads.

### Genome annotation

Genes were identified using Prodigal [36] as part of the Oak Ridge National Laboratory genome annotation pipeline, followed by a round of manual curation using the JGI GenePRIMP pipeline [37]. The predicted CDSs were translated and used to search the National Center for Biotechnology Information (NCBI) nonredundant database, UniProt, TIGRFam, Pfam, PRIAM, KEGG, COG, and InterPro databases. Additional gene prediction analysis and manual functional annotation was performed within the Integrated Microbial Genomes Expert Review (IMG-ER) platform [38].

## Genome properties

**Figure 3:**
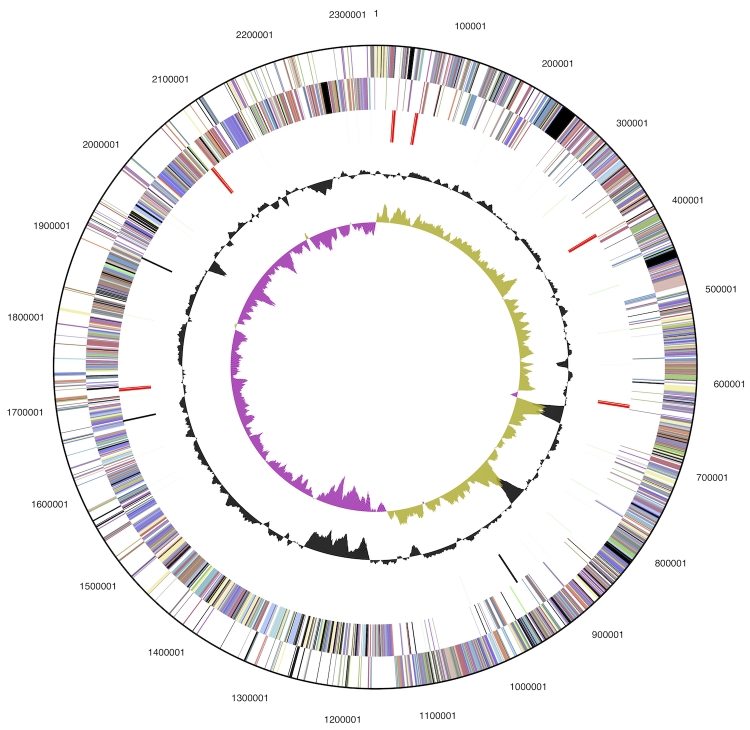
Graphical circular map of the genome. From outside to the center: Genes on forward strand (color by COG categories), Genes on reverse strand (color by COG categories), RNA genes (tRNAs green, rRNAs red, other RNAs black), GC content, GC skew.

The genome is 2,329,769 bp long and comprises one circular chromosome with a 55.8% GC content (Table 3 and Figure 3). Of the 2,182 genes predicted, 2,101 were protein-coding and 81 were RNAs; 75 pseudogenes were also identified. The majority of protein-coding genes (75.3%) were assigned a putative function while the remaining ones were annotated as hypothetical proteins. The properties and the statistics of the genome are summarized in Table 3. The distribution of genes into COGs functional categories is presented in Table 4.

**Table 3:** Genome Statistics

**Attribute**	**Value**	**% of Total**
Genome size (bp)	2,329,769	100.00%
DNA coding region (bp)	2,096,198	89.97%
DNA G+C content (bp)	1,301,006	55.84%
Number of replicons	1	
Extrachromosomal elements	0	
Total genes	2,182	100.00%
RNA genes	81	3.71%
rRNA operons	6	
Protein-coding genes	2,101	96.29%
Pseudo genes	75	3.44%
Genes with function prediction	1,642	75.25%
Genes in paralog clusters	283	13.11%
Genes assigned to COGs	1,661	76.12%
Genes assigned Pfam domains	1,724	79.01%
Genes with signal peptides	361	16.54%
Genes with transmembrane helices	519	23.79%
CRISPR repeats	2	

**Table 4:** Number of genes associated with the general COG functional categories

**Code**	**Value**	**%age**	**Description**
J	139	6.6	Translation, ribosomal structure and biogenesis
A	0	0.0	RNA processing and modification
K	128	6.1	Transcription
L	119	5.7	Replication, recombination and repair
B	1	0.0	Chromatin structure and dynamics
D	21	1.0	Cell cycle control, mitosis and meiosis
Y	0	0.0	Nuclear structure
V	37	1.8	Defense mechanisms
T	62	3.0	Signal transduction mechanisms
M	110	5.2	Cell wall/membrane biogenesis
N	7	0.3	Cell motility
Z	0	0.0	Cytoskeleton
W	0	0.0	Extracellular structures
U	38	1.8	Intracellular trafficking and secretion
O	55	2.6	Posttranslational modification, protein turnover, chaperones
C	114	5.4	Energy production and conversion
G	72	3.4	Carbohydrate transport and metabolism
E	204	9.7	Amino acid transport and metabolism
F	58	2.8	Nucleotide transport and metabolism
H	79	3.8	Coenzyme transport and metabolism
I	47	2.2	Lipid transport and metabolism
P	92	4.4	Inorganic ion transport and metabolism
Q	16	0.8	Secondary metabolites biosynthesis, transport and catabolism
R	240	11.4	General function prediction only
S	145	6.9	Function unknown
-	521	24.8	Not in COGs

## Insights from the genome sequence

Different from most organisms for which the type strain genomes have so far been described in the GEBA series, *A. fermentans* strain VR4^T^ is biochemically well described. The strain has been intensively studied for many years. Here, we describe the genomic location of the genes for the biochemically characterized enzymes, as well as the annotation of the genome using bioinformatic approaches, which may reveal additional physiological properties of the organism.

### Glutmate fermentation via 2-hydroxyglutarate

The ability of *A. fermentans* to use amino acids as the sole energy source for growth is a well known characteristic, with glutamic acid being the most important amino acid for the organism [1,4]. Strain VR4^T^ ferments glutamate *via* the 2-hydroxyglutarate pathway, in which, glutamate is converted to a key intermediate – (R)-2-hydroxyglutaryl-CoA, which is dehydrated to glutaconyl-CoA, followed by decarboxylation to crotonyl-CoA, then to ammonia, CO_2_, acetate, butyrate and hydrogen. An unusual dehydratase contains an [4Fe–4S]^2+^ cluster – acting as an activator or initiator of dehydration, is activated by an ATP-dependent one-electron reduction [29,39,40]. The extra energy produced is conserved via ΔμNa^+^ generated by the decarboxylation of glutaconyl-CoA [41].

The dehydratase system of strain VR4^T^ consists of two oxygen-sensitive protein components: component A – the activator (*HgdC*) and component D – the actual dehydratase (*HgdAB*) [29,40]. Component A has been crystallized and its structure has been determined by X-ray crystallography at 3 Å resolution [42].

The glutaconyl-CoA decarboxylase of *A. fermentans* is a biotin-dependent sodium pump, consisting of three major polypeptide subunits: biotin carrier (alpha, gcdA), carboxytransferase (beta, gcdB) and carboxylase, the actual sodium pump (gamma, gcdC) [43]. There is additional small subunit (delta, gcdC), whose function is unclear [43]. Glutaconate CoA-transferase consists of two different polypeptide chains and is necessary for the decarboxylation of glutaconate [44].

**Figure 4:**
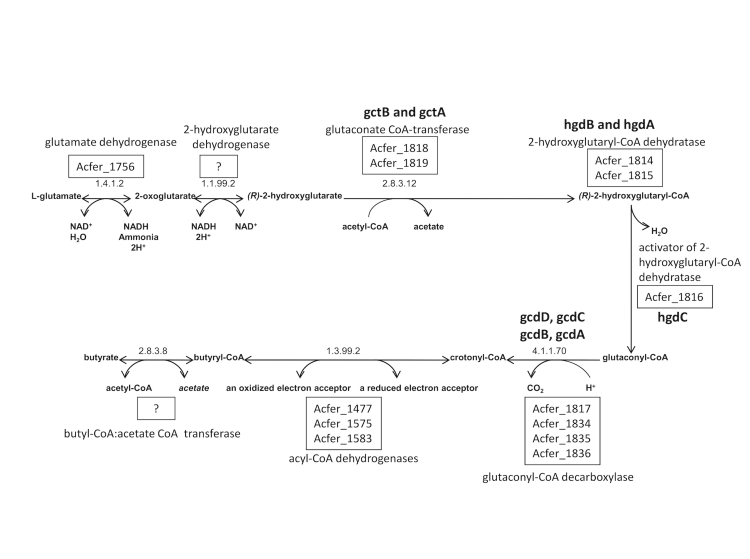
MetCyc pathway [46] along with the gene locus names, representing the enzymes identified in the pathway.

The hydroxyglutarate operon has been experimentally studied [45] and all encoding genes are annotated in the genome (Figure 4)

The entire 7.9 kb long gene cluster, consists of 2-hydroxyglutaryl-CoA dehydratase D-components *hgdAB* (Acfer_1814 and 1815), CoA-substrate-specific enzyme activase (Acfer_1816), glutaconyl-CoA decarboxylase α subunit *gcdA* (Acfer_1817), glutaconate CoA-transferase α− and β−subunits (*gctAB*, Acfer_1819 and 1818). The glutaconyl-CoA decarboxylase β−, γ− and δ-subunits: *gcdB*, *gcdC* and *gcdD* (Acfer_1834, Acfer_1835 and Acfer_1836) are encoded nearly 15kb upstream from this operon, forming a second operon [43].

In addition to the above-mentioned protein complexes, the gene encoding glutamate dehydrogenase (NAD(P)(+)) (Acfer_1756) is encoded at the beginning of the gene cluster. Three acyl-CoA dehydrogenase genes (Acfer_1477, Acfer_1575 and Acfer_1583) were annotated at various locations, completing the pathway. Nevertheless, genes encoding 2-hydroxyglutarate dehydrogenase and Butyl-CoA:acetate CoA transferase have not yet been identified. Possibly these enzymes have additional functions in other pathways and have been annotated distinctly.

**Figure 5:**
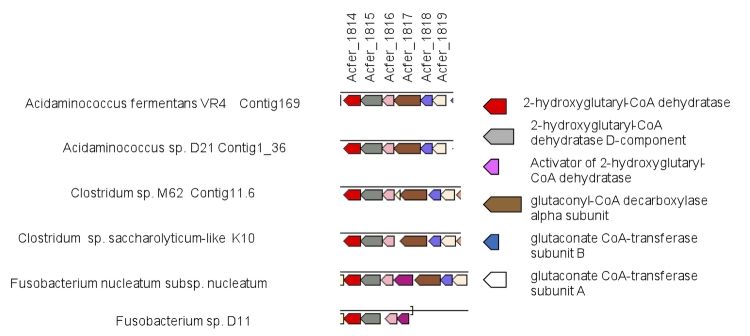
Gene ortholog neighborhoods of hydroxyglutarate operons Colors indicate ortholog groups. The size of the bar approximately corresponds to gene size. Date are taken from [47]

Figure 5 shows a comparison of the hydroxyglutarate operon among various organisms. The positional gene cluster is conserved in the two strains belonging to the genus *Acidaminococcus* (*A. fermentans* strain VR4^T^ and *A. intestini* strain D21), as well as in the two clostridia, whereas *Fusobacterium* differ slightly.

### Overview on transport systems

About 310 putative transporter genes are annotated in the genome of strain VR4^T^, which constitute roughly 15% of the coding genes. The majority of these transporters belong to two groups: secondary carriers and the ATP-binding cassette ABC-type carriers. The most frequent class of ABC-type transport proteins are for importing iron/metal ions and amino acids, as shown in Table 5.

**Table 5:** Overview of the ABC-type transporters within the genome of *A. fermentans* VR4^T^

**ABC-type transporter**	**complete system/genes**	**incomplete system/genes**
ABC-type organic nutrient uptake systems --ABC amino acid transporter --ABC spermidine/putrescine transporter	3 /13 1 /4	1 /1 0 /0
		
ABC-type inorganic nutrient uptake systems --ABC iron uptake and other metal ion transporter -- ABC nitrate/sulfonate/bicarbonate transporter -- ABC phosphate transporter	9 /29 1 /3 2 /5	3 /3 0 /0 0 /0
		
ABC-type efflux pumps -- ABC multidrug transporter -- ABC lipid A exporter -- ABC antimicrobial peptide transporter	4 /9 0 /0 1 /2	1 /1 1 /1 0 /0
		
uncharacterized ABC-type systems	2 /4	3 /3
		

The numbers represent the number of the ABC system or the number of the genes involved, respectively. A functional ABC system, containing all necessary subunits, is referred to as complete system, otherwise it is labeled as incomplete.

Among all the ABC-type transport systems, 41% are related to the transport of iron or other metal ions, comprising the largest ABC transporter group annotated. Additionally, two ferrous iron uptake (FeoB) systems (5 genes; TC#9.A.8) were annotated, which are involved in G protein coupled Fe^2+^ transport. At least 15 other gene products are involved in iron or magnesium transport and heavy metal transport and detoxification. Presumably, *Acidaminococcus* has adapted the ability to sequester iron from the host as a survival strategy. The abundance of this particular group of transporter genes might suggest diverse mechanisms evolved in order to compete for the limited iron supply in the gastrointestinal environment.

The second most abundant ABC transporter group consists of amino acid transporters, followed by multi drug or antimicrobial efflux pumps (Table 5). This trend is visible in secondary carrier proteins; more than 18 and 26 genes encoded for amino acid transport and multi drug or antimicrobial efflux pumps, respectively. At least six genes are annotated as encoding a Na^+^:glutamate symporter (TC# 2.A.27.1). This corroborates the most prominent physiological characteristic of the organism, namely that glutamate is the most important energy and carbon source. No functional sugar transport protein was identified, indicating that this organism does not utilize sugar.

Transporters for carboxylate are also noticeable. For example, the tripartite ATP-independent periplasmic transporter (TRAP-T) family (TC# 2.A.56) is involved in the uptake of widely divergent compounds, mostly carboxylate derivatives [48]. Five TRAP systems are found in the genome, including 12 genes, one TAXI type system and four DctPQM systems. The abundance of the TRAP-T proteins is indicative of the capability of this organism to import carboxylate derivatives such as those produced by the host metabolism or fermentation by rumen microbiota, thus constituting a recycled food web and a beneficial nutritional cycle.

The Bile Acid:Na^+^ symporter (TC# 2.A.28) previously identified in intestinal, liver and kidney tissues of animals is identified at various locations within the genome (Acfer_0208, Acfer_0775 and Acfer_1270). This might be indicate horizontal gene transfer (HGT) between the host and *A. fermentans* VR4^T^. It has been shown that the acquisition of eukaryotic genes in bacteria is frequently the result of a transfer from the host [49]. Given the environmental niche of *A. fermentans*, host-mediated HGT might well have occurred.

No genes for flagellar machinery (TC#3.A.6) are encoded in the genome, which is consistent with the observation of non-motility. *A. fermentans* VR4^_T_^ probably uses a type II secretion (Sec system) for protein secretion, as all components of the Sec protein export system are present (SecA, SecYEG, SecDF), except for SecB, which may be functionally replaced by a different chaperone.

### Comparison with the genome of Acidaminococcus intestini D21

The genome sequence of another member of the genus *Acidaminococcus* (*A. intestini*) which was isolated from an human gastrointestinal tract D21, has been partially deciphered by the Broad Institute. The unfinished yet annotated genome sequence is deposited at NCBI (ACGB00000000) and IMG-GEBA (object-ID 643886056). The 16S rRNA sequence from *A. sp.* D21 differs from the one obtained from *A. intestini* type strain ADV 255.99^T^ (AF473835) by just three nt, but it shares only 95.86 to 96.05% sequence similarity with *A. fermentans* VR4, indicating a considerable evolutionary distance between the two species. Despite these discrepancies, the annotated genomes indicated quite a few common physiological traits.

For instance, the 2-hydroxyglutarate operon was well conserved between the two genomes, including position, structure and individual genes (Fig.5). This suggests that both species have adopted the same glutamate fermentation pathway.

The citrate fermentation via oxaloacetate and pyruvate is another important pathway by which *A. fermentans* VR4 is able to utilize trans-aconitate and citrate as an energy source [4,28]. Genes responsible for this processing tend to cluster in both genomes. Unlike the case of glutamate fermentation, genes within the trans-aconitate and citrate fermentation pathway exhibit a distinct organization in the two genomes (data not shown). This might imply differences in gene regulation or in substrate uptake. Table 6 lists major genes identified from the fermentation pathways discussed above.

**Table 6:** A list of genes discussed, reflecting the organism’s physiological insights

Enzymes	Locus	Gene annotation
**From the glutamate fermentation pathway**		
Acfer_1756		Glutamate dehydrogenase
		
Acfer_1818		glutaconate CoA-transferase β subunit
Acfer_1819		glutaconate CoA-transferase α subunit
		
Acfer_1814		2-hydroxyglutaryl-CoA dehydratase (β subunit)
Acfer_1815		2-hydroxyglutaryl-CoA dehydratase (subunit α)
		
Acfer_1816		2-hydroxyglutaryl-CoA dehydratase activator protein
		
Acfer_1817		glutaconyl-CoA decarboxylase subunit α
Acfer_1834		glutaconyl-CoA decarboxylase β subunit
Acfer_1835		glutaconyl-CoA decarboxylase subunit r
Acfer_1836		glutaconyl-CoA decarboxylase sodium pump, subunit r
		
Acfer_1477		acyl-CoA dehydrogenase domain protein
Acfer_1575		acyl-CoA dehydrogenase domain protein
Acfer_1583		acyl-CoA dehydrogenase domain protein
**Enzymes of β-lactamase and the related**		
		
Acfer_0250		β-lactamase domain-containing protein
Acfer_0522		Zn-dependent hydrolase of the β-lactamase fold
Acfer_0551		RNA-metabolizing metallo-β-lactamase
Acfer_0879		β-lactamase class A-like
Acfer_1020		RNA-metabolizing metallo-β-lactamase
Acfer_1231		β-lactamase domain protein
Acfer_1515		β-lactamase domain-containing protein
Acfer_1556		β-lactamase class A-like
Acfer_1591		β-lactamase class C
**From fermentation of trans-aconitate via** **citrate, oxaloacetate and pyruvate**		
Acfer_0075		pyruvate flavodoxin/ferredoxin oxidoreductase domain protein
		
Acfer_0096		aconitate hydratase domain protein
Acfer_0097		citrate transporter
		
Acfer_0406		citrate transporter
Acfer_0407		citrate transporter
Acfer_0408		citrate lyase, α subunit
Acfer_0409		citrate lyase, β subunit
Acfer_0410		citrate lyase acyl carrier protein, r subunit
		
Acfer_0489		pyruvate ferredoxin/flavodoxin oxidoreductase
		
Acfer_0606		isocitrate dehydrogenase, NADP-dependent
Acfer_0630		dicarboxylate carrier MatC domain protein
Acfer_0631		aconitate hydratase domain protein
		
Acfer_1070		citrate transporter
		
Acfer_1362		oxaloacetate decarboxylase; pyruvate carboxyltransferase
		
Acfer_1968		isocitrate/isopropylmalate dehydrogenase
Acfer_1969		dicarboxylate carrier MatC domain protein
Acfer_1971		aconitate hydratase
Acfer_1973		aconitate hydratase domain protein

*Acidaminococcus* has been considered highly susceptible to β-lactam antibiotics until Galán et al. [50] discovered the first β-lactamase in this species. Throughout the genomes of *A. fermentans* VR4 and *A. sp*. D21, there are about 10 β-lactamase or β-lactamase related genes (Table 6). This indicates that both *A. fermentans* VR4 and *A. sp*. D21 can be resistant to β-lactam antibiotics. The organism might thus contribute, *via* HGT, to the origin or spread of resistance genes in one of the most complex microbial ecosystems known.
